# The dysregulated unfolded protein response in diabetic kidney disease: mechanisms and crosstalk with cell death pathways

**DOI:** 10.3389/fphar.2026.1875777

**Published:** 2026-06-24

**Authors:** Hao Zhang, Kaixiang Li, Jia Ma, Ling Ma, Muhammad Asad Farooq, Jing E

**Affiliations:** 1 Department of Nephrology, People’s Hospital of Ningxia Hui Autonomous Region, Yinchuan, China; 2 The First Dongguan Affiliated Hospital, Guangdong Provincial Key Laboratory of Medical Immunology and Molecular Diagnostics, Guangdong Medical University, Dongguan, China; 3 Department of Clinical Medicine, Xi’an Jiaotong University, Xi’an, China

**Keywords:** apoptosis, diabetic kidney disease (DKD), ER stress, ER-phagy, ferroptosis, unfolded protein response (UPR)

## Abstract

Diabetic kidney disease (DKD) is the leading cause of chronic kidney disease and end-stage kidney failure worldwide, yet the molecular mechanisms driving progressive renal injury remain incompletely understood. Chronic metabolic stress in diabetes disrupts endoplasmic reticulum (ER) homeostasis, leading to sustained activation of the unfolded protein response (UPR). While transient UPR signaling is adaptive and restores proteostasis, persistent ER stress in the diabetic milieu shifts UPR signaling toward maladaptive pathways that promote cellular dysfunction and death. This review summarizes the canonical UPR branches mediated by protein kinase RNA-like ER kinase (PERK), inositol-requiring enzyme 1α (IRE1α), and activating transcription factor 6 (ATF6), and discusses their dysregulation in the diabetic kidney. We highlight how chronic glucolipotoxicity, oxidative stress, and protein overload drive prolonged UPR activation in podocytes and tubular epithelial cells, leading to loss of proteostatic balance and progressive nephron injury. Importantly, emerging evidence indicates that UPR signaling interacts with multiple regulated cell death pathways, including apoptosis, autophagy dysfunction, ferroptosis, pyroptosis, and necroptosis, forming a pathological crosstalk network that determines renal cell fate. This maladaptive integration amplifies inflammation, oxidative stress, mitochondrial dysfunction, and fibrotic remodeling, ultimately contributing to podocyte depletion, tubular atrophy, and disease progression. Finally, we discuss therapeutic strategies to restore ER proteostasis and modulate UPR-mediated cell death signaling, highlighting their potential as disease-modifying approaches in DKD. A deeper understanding of UPR dysregulation and its interaction with cell death pathways may provide novel mechanistic insights and facilitate the development of targeted therapies for diabetic kidney disease.

## 1 Introduction

### 1.1 Global burden of diabetic kidney disease

Diabetic kidney disease (DKD) represents one of the most prevalent complications of diabetes mellitus (DM). It is a leading cause of chronic kidney disease (CKD) and end-stage kidney disease (ESKD) worldwide (Thomas et al., 2015; Fenta et al., 2023). With the global incidence of DM rising sharply, the burden of DKD continues to escalate, particularly in middle-income countries where healthcare systems face growing clinical and economic strain. Epidemiological data indicate that over 30% of individuals with diabetes will develop DKD in their lifetime, making it a major driver of dialysis dependence and kidney transplantation (Alicic et al., 2017). Apart from kidney failure, DKD increases the risk for cardiovascular complications and a high mortality rate, which highlights a poor long-term prognosis (Afkarian et al., 2013).

Although several strides have been made to control blood glucose and blood pressure levels, and the inhibition of the angiotensin-aldosterone pathway (Sindhu et al., 2023), yet current treatment options only slow the kidney damage, rather than reversing it or at least stopping the process. Several pharmacological agents, including sodium-glucose cotransporter 2 (SGLT2) inhibitors and nonsteroidal mineralocorticoid receptor antagonists, are in clinical practice and have provided meaningful renal protection, yet a significant “residual risk” remains, as these therapies often fail to address the underlying intracellular collapse that drives disease progression (Ruiz-Ortega et al., 2020; Młynarska et al., 2024; Neuen et al., 2024; Verma et al., 2026). Existing therapies largely target systemic hemodynamic and metabolic factors, but do not sufficiently address the intracellular stress responses and maladaptive signaling networks that ultimately drive renal injury (Xie et al., 2022). Defining these intracellular mechanisms is therefore essential for developing disease-modifying strategies in DKD.

### 1.2 Overview of the unfolded protein response and rationale for UPR cell death crosstalk in DKD

A central feature of diabetes is chronic metabolic stress, which places sustained pressure on cellular homeostatic systems, particularly within the endoplasmic reticulum (ER). The ER is responsible for protein folding, calcium balance, and lipid synthesis, but these functions become compromised under conditions such as hyperglycemia, insulin resistance, lipid accumulation, and inflammation. As a result, misfolded or unfolded proteins accumulate, triggering ER stress (; Byun et al., 2025). In metabolically active tissues such as the kidney, ER stress is not merely a secondary consequence of diabetes but a proximal driver of cellular dysfunction and injury.

In response, cells activate the unfolded protein response (UPR), coordinated by PERK, IRE1α, and ATF6. In DKD models and patient-relevant datasets, transient adaptive signaling is progressively replaced by sustained pro-inflammatory and pro-death programs when ER stress remains unresolved (Kaufman, 2002; Gao et al., 2023).

Although UPR is a well-established feature of DKD, evidence indicates that UPR signaling alone is unable to explain the progression and irreversible nature of renal injury seen in diabetic patients (Wang F. et al., 2025). Increasing evidence points to a more integrated model, where prolonged ER stress engages multiple cell death pathways through complex molecular crosstalk (Hetz and Papa, 2018). Thus, DKD progression is not simply a consequence of ER stress *per se*, but of a failure to resolve proteostasis coupled with activation of downstream death programs. This transition marks the point at which adaptive responses give way to irreversible nephron loss and fibrotic remodeling. Understanding how UPR signaling interfaces with these cell death pathways is, therefore, central to defining the mechanistic basis of DKD progression.

### 1.3 Scope and structure of the review

This review goes beyond a simple description of UPR components and focuses on how they functionally interact with cell-fate decisions in diabetic kidney disease. We begin by outlining the major sources and patterns of ER stress in the diabetic kidney, followed by a brief overview of the three canonical UPR signaling pathways. The main emphasis is placed on the molecular links between UPR activation and different forms of cell death, and how these processes contribute to glomerular and tubular damage. By framing the UPR as a central signaling hub that integrates metabolic stress and the execution of cell death, this review aims to provide a unified mechanistic model of DKD progression. Finally, we address current therapeutic perspectives, identify existing knowledge gaps, and highlight future research directions, with particular attention to UPR-cell death crosstalk as a promising target for disease-modifying strategies in DKD.

**FIGURE 1:**
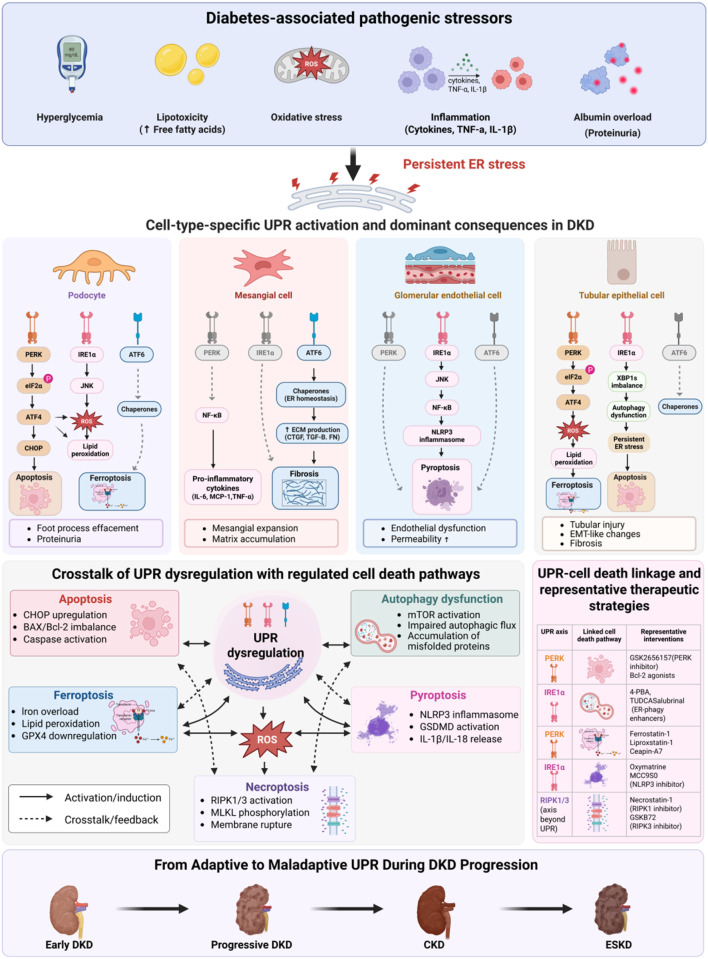
Canonical unfolded protein response pathways are activated in DKD.

**FIGURE 2:**
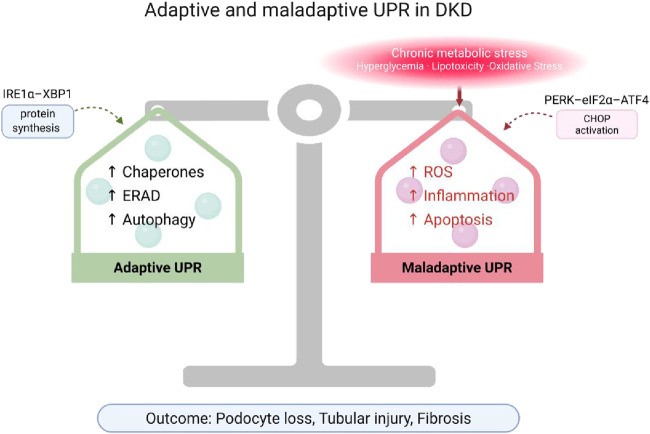
Adaptive and maladaptive UPR in diabetic kidney disease.

Compared with recent DKD reviews that mainly summarize ER stress components or isolated death pathways, this review integrates several advances reported in recent years: branch-resolved UPR maladaptation signatures (persistent PERK-CHOP activation and stress-biased IRE1α outputs) in DKD-relevant settings (Zhang et al., 2023; Cao et al., 2025; Hong and Inagi, 2025), compartment-informed differences between podocyte ferroptotic susceptibility and tubular pyroptotic/necroptotic coupling (Wang et al., 2023; Kang et al., 2024; Lv et al., 2025), and a mechanism-guided translational framework linking proteostasis correction, branch-level modulation, and delivery constraints (Li et al., 2023; Rayego-Mateos et al., 2023; Zhao Y. et al., 2024; Aguilar-Cartes et al., 2026). Accordingly, the novelty of this review is presented as evidence-supported integration from mechanism to intervention logic. Specifically, compared with the Frontiers in Pharmacology review on ER stress in AKI (Cheng et al., 2024) and the Cell Communication and Signaling review on targeting cell death pathways in DKD (Liu et al., 2024), the present review adds branch-resolved maladaptation signatures in DKD renal compartments, cross-compartment death-coupling logic (podocyte versus tubular bias), and a pharmacology-oriented bridge from molecular crosstalk hubs to intervention-layer strategy. An overview of canonical UPR signaling and the adaptive–to–maladaptive transition in DKD is shown in Figures 1, 2.

## 2 The canonical UPR pathways

The UPR is a highly conserved intracellular signaling network that maintains ER homeostasis under conditions of proteotoxic stress. In metabolically active tissues such as the diabetic kidney, chronic stress persistently engages the UPR in an attempt to restore ER function. This response is mediated by three transmembrane sensors (PERK, IRE1α, and ATF6), which operate in parallel and coordinate adaptive and maladaptive outcomes that ultimately shape cell fate. Importantly, these pathways do not function independently but are dynamically integrated, with their combined output defining the cellular proteostatic capacity under stress.

### 2.1 PERK-eIF2α-ATF4 signaling: global translation attenuation versus selective translation

Protein kinase RNA-like endoplasmic reticulum kinase (PERK) acts as an early sensor of ER stress. Upon accumulation of misfolded proteins, it dissociates from the chaperone GRP78, oligomerizes, and undergoes autophosphorylation. Activated PERK then phosphorylates the α subunit of eukaryotic initiation factor 2 (eIF2α), leading to suppression of cap-dependent protein synthesis and reducing the influx of newly synthesized polypeptides into the stressed ER (Rozpedek et al., 2016) This rapid reduction of translation is a key adaptive response that limits further proteotoxic burden.

Despite this global repression, eIF2α phosphorylation selectively enhances the translation of specific stress-responsive mRNAs containing upstream open reading frames, most notably activating transcription factor 4 (ATF4) (Vanhoutte et al., 2021). ATF4 subsequently translocates to the nucleus, where it regulates genes involved in amino acid metabolism, redox balance, and autophagy, thereby supporting cellular adaptation to stress (Zhou et al., 2022). However, this pathway is highly context-dependent. Under prolonged or unresolved ER stress, sustained PERK-ATF4 signaling shifts from a protective to a deleterious program. This shift reflects a transition from adaptive translational control to transcriptional reprogramming toward cell death. Persistent ATF4 activity drives the induction of C/EBP homologous protein (CHOP), which suppresses anti-apoptotic BCL-2 family members, increases oxidative stress, and sensitizes mitochondria to both apoptotic and non-apoptotic cell death pathways (Chen et al., 2014). In this way, the PERK axis functions as a central checkpoint that converts unresolved proteotoxic stress into a pro-death signal.

### 2.2 IRE1α-XBP1s signaling: mRNA splicing and ER-associated degradation

Inositol-requiring enzyme 1α (IRE1α) is the most conserved branch of the UPR and serves as a key integrator of ER stress signals (Liu et al., 2025). Upon activation, IRE1α dissociates from GRP78, dimerizes or oligomerizes, and undergoes trans-autophosphorylation, which enables its endoribonuclease activity. This activity mediates the unconventional splicing of X-box binding protein 1 (XBP1) mRNA, producing the active transcription factor XBP1s (Zhao W. et al., 2024; Liu et al., 2025). XBP1s drives a transcriptional program that expands the ER’s folding capacity by inducing the expression of molecular chaperones, foldases, and lipid biosynthetic enzymes required for membrane expansion. At the same time, it enhances ER-associated degradation (ERAD), facilitating the clearance of terminally misfolded proteins through retrotranslocation and proteasomal degradation. Together, these processes restore proteostasis and support cell survival during transient stress.

With sustained or excessive stress, however, IRE1α signaling becomes dysregulated. Hyperactivation of its RNase function initiates regulated IRE1-dependent decay (RIDD), leading to degradation of selected mRNAs and microRNAs. Through degradation of survival-associated transcripts, RIDD shifts IRE1α signaling from adaptive proteostasis toward cellular dysfunction. This shift disrupts protein homeostasis, promotes metabolic imbalance, and amplifies inflammatory and pro-apoptotic signaling. In parallel, IRE1α engages stress kinase pathways through TRAF2, further linking ER stress to inflammatory and death signaling. Thus, similar to PERK, the IRE1α-XBP1s axis exhibits a functional switch, with its outcome determined by the intensity and duration of ER stress.

### 2.3 ATF6 signaling: proteolytic activation and chaperone upregulation

Activating transcription factor 6 (ATF6) constitutes a distinct branch of UPR that primarily enhances the ER’s folding and quality control capacity (Coleman et al., 2018; Wadgaonkar et al., 2022). Under basal conditions, ATF6 is retained in the ER membrane through its association with GRP78 (Madhusudhan et al., 2015; Liu et al., 2025). During ER stress, it is released and trafficked to the Golgi apparatus, where sequential cleavage by site-1 and site-2 proteases generates an active N-terminal fragment that translocates to the nucleus (Velho et al., 2017). Once activated, ATF6 induces a broad transcriptional program that includes molecular chaperones such as GRP78 and GRP94, protein disulfide isomerases, and components of the ERAD machinery (Hwang and Qi, 2018). These responses collectively enhance protein folding, promote clearance of misfolded proteins, and facilitate recovery from stress.

Compared with PERK and IRE1α, ATF6 signaling is generally considered more adaptive. However, its role is still context dependent. In the diabetic kidney, the failure of the ATF6 branch to keep pace with metabolic demand creates a “insufficient proteostatic capacity”, leaving renal cells vulnerable to the more destructive outputs of PERK and IRE1α. This relative insufficiency disrupts the balance between adaptive and maladaptive UPR outputs. Insufficient or delayed activation can impair chaperone induction and prolong ER stress, while sustained activation may intersect with inflammatory and metabolic pathways, indirectly influencing cell fate (Lu et al., 2025). Evidence from stress-resistance biology also supports a protective baseline role for ATF6-linked programs under chronic stress contexts^(48)^. In this way, ATF6 helps determine whether ER stress is resolved or progresses toward dysfunction.

### 2.4 ER proteostasis: balancing folding capacity and demand

ER proteostasis reflects the dynamic balance between the cellular demand for protein synthesis and the ER’s capacity to correctly fold, process, and traffic proteins. This balance is particularly critical in metabolically active and secretory cells, where protein load is high (Urra et al., 2024). It is maintained through an integrated system of molecular chaperones, folding enzymes, ER-associated degradation, and translational control mechanisms, coordinated by the UPR (Hwang and Qi, 2018). Through coordinated signaling via PERK, IRE1α, and ATF6, cells adapt to increased protein load by transiently reducing translation, expanding folding capacity, and enhancing degradation of misfolded proteins (Huang et al., 2019).

Under acute or transient stress, these mechanisms are typically sufficient to restore ER function. In contrast, chronic metabolic stress, such as that seen in diabetes, imposes a sustained burden through hyperglycemia, lipotoxicity, oxidative stress, and inflammation. Over time, this overwhelms the proteostatic network, resulting in prolonged UPR activation and a gradual shift toward maladaptive signaling. This is characterized by persistent translational repression, redox imbalance, heightened inflammatory responses, and activation of multiple cell death pathways. Importantly, this state reflects not only increased stress intensity but a failure of resolution mechanisms, locking cells into a chronic stress, adapted phenotype. Failure to re-establish proteostasis can create a self-perpetuating cycle of stress and injury, ultimately driving progressive tissue damage. In this context, disruption of ER proteostasis represents a central mechanism linking chronic metabolic stress to organ dysfunction in DKD.

Diabetic stressors, including glucolipotoxicity and albumin overload, disrupt ER homeostasis and activate three resident sensors (ATF6, PERK, and IRE1α) in both podocytes and tubular epithelial cells. Upon ER stress, ATF6 translocates to the Golgi for proteolytic activation to enhance folding capacity, although this adaptive buffering may become insufficient under prolonged metabolic load. PERK phosphorylates eIF2α to attenuate global translation while promoting ATF4 and its downstream pro-apoptotic target CHOP. IRE1α mediates unconventional XBP1 mRNA splicing to generate adaptive XBP1s, whereas persistent overactivation can engage RIDD and JNK signaling. Recent DKD evidence supports mapping sustained UPR stress outputs to compartment-biased death phenotypes, including mitochondrial apoptosis and ferroptotic susceptibility in podocytes, and inflammasome-pyroptotic and necroptotic signaling in tubular compartments (Kang et al., 2024; Cao et al., 2025; Hong and Inagi, 2025; Huang et al., 2025; Lv et al., 2025).

## 3 Mechanisms of UPR dysregulation in the DKD

Chronic metabolic stress in diabetes persistently disrupts ER homeostasis, driving sustained and dysregulated UPR signaling. Rather than resolving proteotoxic stress, prolonged exposure to glucotoxicity, lipotoxicity, and advanced glycation end-products (AGEs) pushes the UPR toward a maladaptive state (Ahmad et al., 2022; Wang and Zhang, 2024; Zuo et al., 2024). This persistent maladaptive activation progressively impairs the cellular machinery required for renal filtration and reabsorption. While early insights into UPR dysregulation come largely from pancreatic β-cells, the diabetic kidney presents a distinct context. Here, ER stress arises from the combined burden of metabolic overload, lipid accumulation, oxidative stress, and protein handling, creating a positive feedback cycle of injury (Madhusudhan et al., 2015; Liu et al., 2025). The key issue is not simply UPR activation, but the failure to resolve ER stress, which shifts signaling from adaptive to pathogenic.

### 3.1 Chronic metabolic overload

Renal cells in DKD are continuously exposed to hyperglycemia, elevated free fatty acids, and increased intratubular protein load, generating combined glucotoxic, lipotoxic, and proteotoxic stress (Khan et al., 2026). Hyperglycemia promotes the formation of AGEs, reactive oxygen species (ROS), and extracellular matrix proteins, increasing the burden on the ER folding machinery (Jung and Yoo, 2022). In parallel, albuminuria drives excessive protein reabsorption in tubular cells, further overwhelming ER capacity and promoting the accumulation of misfolded proteins.

Lipotoxicity adds a structural dimension to ER stress. Saturated fatty acids such as palmitate incorporate into ER membranes, altering lipid composition, reducing membrane fluidity, and impairing chaperone function and calcium homeostasis (McNally et al., 2022). These changes, together with lipid-induced oxidative stress, exacerbate ER dysfunction and act synergistically with glucotoxicity (Cao et al., 2023). Related inflammatory-ER stress coupling has also been reported in vascular diabetic complications through the C5a-C5aR1-PERK-eIF2α-ATF4 axis. Thus, ER stress in DKD reflects both increased protein load and intrinsic disruption of ER structure and function.

Under these conditions, PERK, IRE1α, and ATF6 remain chronically activated. In the absence of a recovery phase, their signaling becomes dysregulated: sustained PERK activity promotes ATF4 and CHOP-driven apoptosis; IRE1α shifts from adaptive XBP1 splicing toward activation of c-Jun N-terminal kinase (JNK) and Nuclear factor kappa-light-chain-enhancer of activated B cells (NF-κB) pathways; and ATF6, although initially protective, becomes insufficient to restore proteostasis (Wang et al., 2022). Impaired autophagy, persistent oxidative stress, and reduced XBP1 activity further prevent the resolution of ER stress.

This unresolved stress has cell-specific consequences. In podocytes, it disrupts cytoskeletal integrity and promotes apoptosis, leading to barrier dysfunction and proteinuria. In tubular epithelial cells, it drives inflammation and fibrosis. Together, these processes establish a positive feedback cycle in which metabolic overload sustains ER stress and maladaptive UPR signaling.

### 3.2 Podocyte-specific UPR dysregulation in DKD

Podocytes are highly specialized, terminally differentiated cells that play a critical role in maintaining the integrity of the glomerular filtration barrier (Ouyang et al., 2025). Their unique structural and functional features render them particularly vulnerable to ER stress due to their high dependence on precise protein folding and limited regenerative capacity (Fan et al., 2017). In the diabetic milieu, sustained UPR activation progressively shifts toward a pro-apoptotic program.

Chronic IRE1α signaling recruits Tumor necrosis factor receptor-associated factor 2 (TRAF2) and activates JNK, promoting stress-induced apoptosis, while prolonged PERK-eIF2α-ATF4 signaling induces CHOP, suppressing anti-apoptotic B-cell lymphoma 2(Bcl-2) family proteins (Liu et al., 2023). At the same time, impaired ATF6 signaling limits chaperone induction, reducing folding capacity and allowing misfolded proteins to accumulate (Madhusudhan et al., 2015; Takasugi et al., 2023). Collectively, these alterations result in a loss of proteostatic control, leading to podocyte apoptosis, foot process effacement, and proteinuria, which are central features of DKD progression.

### 3.3 Tubular epithelial cell UPR dysfunction in DKD

Proximal tubular epithelial cells are highly metabolically active and continuously exposed to filtered proteins, making them especially susceptible to ER stress. In DKD, chronic albumin overload, hyperglycemia, and lipotoxicity converge to drive sustained UPR activation.

Excessive protein reabsorption increases the folding burden within the ER, while prolonged activation of PERK and IRE1α shifts signaling toward injury. IRE1α enhances NF-κB-mediated inflammatory responses, whereas sustained PERK signaling promotes oxidative stress and apoptotic susceptibility (Eleftheriadis et al., 2023).

UPR dysfunction also contributes directly to tubulointerstitial fibrosis (Mori et al., 2021). Persistent ER stress induces profibrotic mediators such as transforming growth factor-β (TGF-β) and promotes epithelial-mesenchymal transition (EMT)-like phenotypic changes (Nakatsuka et al., 2024). Crosstalk with mitochondrial dysfunction and impaired autophagy further amplifies injury, leading to tubular atrophy, interstitial inflammation, and extracellular matrix accumulation (Rowland and Voeltz, 2012). These changes position tubular UPR dysregulation as a major driver of disease progression.

Compared with podocytes, tubular compartments more frequently exhibit inflammatory death coupling, including CHOP-TXNIP-NLRP3-associated pyroptotic signaling and RIPK3-linked necroptotic injury programs in diabetic settings (Cao et al., 2025; Huang et al., 2025; Li et al., 2025; Liu et al., 2025; Wang Y. et al., 2025).

### 3.4 Imbalance between adaptive and maladaptive UPR in DKD

The UPR is inherently adaptive, restoring ER homeostasis through coordinated regulation of protein synthesis, folding, and degradation (He et al., 2026). In DKD, however, persistent metabolic stress disrupts this balance. Adaptive signaling, particularly the IRE1α-XBP1 axis, is progressively weakened, while pro-apoptotic pathways such as PERK-ATF4-CHOP are amplified (Cao et al., 2025). This imbalance reflects a functional uncoupling of UPR outputs. Reduced XBP1 activity limits folding capacity and exacerbates ER stress, whereas sustained CHOP induction drives apoptosis through mitochondrial dysfunction, oxidative stress, and suppression of survival pathways. At the same time, prolonged IRE1α activation shifts toward JNK and NF-κB signaling, reinforcing inflammation and cell death (Lerner et al., 2012). These changes closely align with key pathological features of DKD, including podocyte loss, tubular injury, and fibrosis.

At the gene-program level, transition to maladaptation tracks with persistent PERK-eIF2α-ATF4/CHOP activation, IRE1α stress-bias signaling, and failure of resolution checkpoints, shifting transcription toward inflammatory and death-associated outputs rather than proteostasis recovery (Yu et al., 2023; Kang et al., 2024; Cao et al., 2025; Huang et al., 2025; Liu et al., 2025).

Importantly, this shift is sustained by defective resolution mechanisms. Impaired autophagy, mitochondrial dysfunction, and disrupted ER-organelle crosstalk prevent recovery of proteostasis, locking cells into chronic stress signaling (Efiong et al., 2024). In this state, the UPR no longer protects the cell but instead actively drives disease progression. Restoring this balance has clear therapeutic implications. Strategies that enhance adaptive signaling, such as boosting XBP1 activity, or limit maladaptive outputs, such as CHOP induction, show promise in mitigating ER stress–mediated injury and slowing DKD progression.

Chronic metabolic stress in diabetes, including hyperglycemia, lipotoxicity, and oxidative stress, disrupts ER homeostasis and activates the UPR. In its adaptive phase, UPR signaling, primarily mediated by the IRE1α-XBP1 axis, enhances protein folding capacity by upregulating molecular chaperones, promotes ER-associated degradation, and stimulates autophagy, thereby restoring proteostasis and supporting cell survival. However, persistent or unresolved ER stress shifts UPR signaling toward a maladaptive state characterized by activation of the PERK-eIF2α-ATF4 pathway and CHOP induction. This transition promotes oxidative stress, inflammation, and apoptosis, leading to cellular injury. The imbalance between adaptive and maladaptive UPR contributes to podocyte loss, tubular epithelial cell damage, and progressive renal fibrosis, ultimately driving the progression of diabetic kidney disease.

## 4 UPR and cell death pathways crosstalk in DKD

In DKD, persistent metabolic stress sustains activation of the endoplasmic reticulum stress response. Although the UPR initially acts to restore proteostasis, prolonged activation drives a shift from adaptation to maladaptation. As ER stress remains unresolved, UPR signaling increasingly intersects with pathways governing inflammation and cell death, marking a key transition in disease progression. This crosstalk is not incidental, but a coordinated transcriptional reprogramming in which stress sensors shift from maintenance to execution of injury.

This shift reflects a critical transition phase at which adaptive outputs, such as XBP1s-mediated protein folding, are no longer sufficient. Instead, UPR sensors engage pro-death signaling through defined intermediates (Chang et al., 2018). For instance, the IRE1α-TRAF2 complex activates JNK and NF-κB, linking ER stress to inflammatory and apoptotic pathways. In parallel, CHOP suppresses pro-survival factors such as Bcl-2 while promoting oxidative stress. Disruption of ER calcium homeostasis further amplifies this crosstalk by driving mitochondrial calcium overload, caspase activation, and inflammasome signaling (Huang et al., 2026).

Collectively, sustained UPR activation dictates renal cell fate, particularly in podocytes and tubular epithelial cells. This dysregulated interplay gives rise to apoptosis, pyroptosis, ferroptosis, and necroptosis, which collectively drive renal injury.

### 4.1 Apoptosis: the UPR-mitochondrial nexus

Apoptosis is a tightly regulated form of programmed cell death characterized by cytoplasmic condensation, pyknosis, and controlled DNA fragmentation, culminating in the formation of apoptotic bodies (Tabas and Ron, 2011). Under physiological conditions, apoptosis is largely immunologically silent due to the externalization of phosphatidylserine, which promotes efficient clearance by phagocytes. In DKD, however, the sustained rate of apoptosis in podocytes and tubular epithelial cells can exceed clearance capacity, resulting in defective efferocytosis, secondary necrosis, and the release of pro-inflammatory intracellular contents. In this context, apoptosis shifts from a homeostatic process to a contributor to inflammation and fibrosis.

At the molecular level, apoptosis is executed by caspases activated through two interconnected pathways. The extrinsic pathway is initiated by death receptors such as Fas and tumor necrosis factor receptor 1 (TNFR1), while the intrinsic pathway is triggered by intracellular stress signals, including oxidative stress and proteotoxic imbalance. In DKD, the intrinsic pathway predominates, reflecting chronic metabolic and ER stress. The ER and mitochondria function as a coupled death axis in the diabetic kidney; once the ER can no longer manage the protein load, it transmits stress signals to mitochondria via calcium flux and ROS. These signals converge on the mitochondria, where mitochondrial outer membrane permeabilization, regulated by Bcl-2 family proteins, leads to cytochrome c release, apoptosome formation, and caspase activation (Zhang et al., 2023). A key layer linking ER stress to apoptosis is the mitogen-activated protein kinase (MAPK) network, which functions as a context-dependent regulator of cell fate (Cybulsky, 2010). The MAPK family, comprising extracellular signal-regulated protein kinases one and 2 (ERK1/2), JNK, and p38 MAPK, exhibits functional specificity depending on the nature and duration of the stimulus. ERK1/2 generally supports adaptive responses, whereas JNK and p38 promote apoptosis under stress conditions (Xue et al., 2020). In DKD, chronic metabo (Huang et al., 2025; Liu et al., 2025) lic and oxidative stress skews this balance toward JNK/p38 activation. Importantly, MAPK signaling is tightly integrated with the UPR (Cybulsky, 2010). Recent DKD-focused evidence supports ER stress-driven apoptotic predominance in renal cells, including UPR-associated mitochondrial injury coupling (Cao et al., 2025).

Mechanistically, persistent ER stress activates IRE1α and PERK, both of which converge on MAPK pathways. IRE1α recruits TRAF2 and activates apoptosis signal-regulating kinase 1 (ASK1), leading to phosphorylation of JNK and p38 MAPK. This IRE1α-TRAF2-ASK1 axis serves as a critical bridge linking UPR signaling to pro-apoptotic and inflammatory outputs in renal cells (Urano et al., 2000). PERK further reinforces this shift by enhancing oxidative stress and downstream transcriptional responses. Although ERK1/2 may transiently support adaptive survival signaling, this protective arm is often suppressed under diabetic conditions. As a result, JNK/p38 pathways dominate, creating a feed-forward loop in which hyperglycemia and lipotoxicity amplify both ER stress and apoptotic signaling (Yu et al., 2023; Xie et al., 2024; Cao et al., 2025). In diabetic nephropathy models, attenuation of p38 MAPK is also associated with reduced inflammatory injury (Huang et al., 2025).

The transition to apoptosis is consolidated at the transcriptional level by the PERK-eIF2α-ATF4 axis, primarily through CHOP induction. CHOP acts as a central executional regulator, repressing anti-apoptotic genes such as Bcl-2 while inducing pro-apoptotic mediators including Bcl-2-associated X protein (Bax), Bcl-2 antagonist 1 (Bak), and P53 upregulated modulator of apoptosis (PUMA), thereby shifting the balance toward mitochondrial permeabilization (Tabas and Ron, 2011; Zhang et al., 2023; Cao et al., 2025). In parallel, CHOP induces ER oxidase 1α (ERO1α), increasing oxidative stress within the ER and further destabilizing mitochondrial integrity. This coupling ensures that unresolved ER stress leads to irreversible apoptotic commitment.

At the pharmacological level, branch-oriented preclinical studies frequently use PERK-focused tools (for example, GSK2656157) to suppress CHOP-biased pro-apoptotic output; however, current DKD evidence should still be interpreted as hypothesis-generating rather than practice-changing ^(103)^.

Beyond transcriptional control, apoptosis in DKD is strongly influenced by ER–mitochondrial crosstalk at mitochondria-associated ER membranes (MAMs). These contact sites regulate calcium transfer, which is essential for cellular homeostasis. Under chronic ER stress, dysregulated UPR signaling enhances activation of ER calcium channels, such as inositol 1,4,5-trisphosphate receptor (IP3R), leading to excessive mitochondrial calcium uptake. This results in the opening of mitochondrial permeability transition pores, membrane depolarization, and the release of pro-apoptotic factors. This mechanism provides a direct link between ER dysfunction and mitochondrial apoptosis, particularly in podocytes, which have limited regenerative capacity. Within this axis, Bcl-2 functions as a critical checkpoint. In addition to inhibiting mitochondrial permeabilization, Bcl-2 localizes to the ER, where it regulates calcium homeostasis and modulates ER stress signaling (Axten et al., 2013; Xu et al., 2020; Zhang et al., 2023). In DKD, PERK–CHOP signaling suppresses Bcl-2 expression while promoting pro-apoptotic proteins such as Bax, thereby facilitating apoptosis in podocytes (Tabas and Ron, 2011). Pharmacological studies further support this axis: agents such as wogonin restore Bcl-2 activity, reduce apoptosis, and preserve glomerular structure in DKD models (Liu et al., 2022). Notably, Bcl-2 may also stabilize ER calcium dynamics and dampen CHOP-driven stress signaling, highlighting its dual role in maintaining both mitochondrial and ER homeostasis. This positions the Bcl-2-UPR interface as a key regulatory node and a potential therapeutic target in DKD (Chen et al., 2014).

### 4.2 Autophagy and ER-Phagy: the proteostatic protective mechanism

Autophagy is a conserved catabolic process that degrades and recycles damaged organelles, misfolded proteins, and excess macromolecules, thereby preserving intracellular homeostasis under stress. It is activated by diverse stimuli, including nutrient deprivation, hypoxia, oxidative stress, and hyperglycemia. In DKD, autophagy functions as a critical quality control system, counteracting the proteotoxic and organelle damage generated by chronic metabolic overload and sustained ER stress (2021).

Autophagy is tightly regulated by interconnected signaling pathways, including the MAPK cascade (Wagner and Nebreda, 2009). In particular, JNK promotes autophagy by phosphorylating Bcl-2, disrupting the Beclin-1-Bcl-2 complex, and enabling autophagosome initiation. Given that Beclin-1 is a core component of the autophagic machinery, this step represents a key regulatory switch. In the diabetic kidney, activation of JNK and p38 MAPK can initially enhance autophagic clearance, serving as an adaptive response to metabolic and oxidative stress (Abo El- Nasr et al., 2022).

This adaptive response is closely integrated with the UPR. Persistent ER stress activates sensors such as IRE1α, which recruits TRAF2 and engages JNK signaling. The resulting IRE1α-JNK axis provides a direct mechanistic link between ER stress and the induction of autophagy, facilitating the clearance of misfolded proteins and damaged ER through a process increasingly recognized as ER-phagy (Jin et al., 2024). In this setting, autophagy acts as a buffering mechanism that complements the UPR to restore proteostasis.

However, this crosstalk is highly context-dependent. In early DKD, autophagy is generally upregulated and protective. With sustained hyperglycemia and lipotoxicity, autophagic flux becomes impaired. This “impaired autophagic flux” turns a recycling system into a accumulation of undegraded cellular substrates, which further fuels ER stress in a self-amplifying pathogenic loop. This impairment is not due to reduced initiation alone but often reflects defective autophagosome-lysosome fusion or degradation, leading to accumulation of undegraded substrates. Such “impaired autophagic flux” exacerbates proteotoxic stress, promotes mitochondrial dysfunction, and further amplifies UPR signaling, particularly along the PERK-CHOP axis. Podocytes, which rely heavily on basal autophagy due to their post-mitotic nature, are especially vulnerable to this failure, resulting in progressive cell loss.

A central regulator of this shift is the mammalian target of rapamycin (mTOR) pathway, which functions as a key negative regulator of autophagy. Under diabetic conditions, chronic activation of mTORC1 inhibits autophagy by phosphorylating ULK1, thereby suppressing its initiation. This establishes a feed-forward loop in which impaired autophagy increases ER stress, while sustained ER stress reinforces mTORC1 activity, in part by suppressing AMP-activated protein kinase (AMPK) signaling. Disrupting this cycle has shown therapeutic potential. Agents such as mangiferin restore autophagic flux via the AMPK-mTOR-ULK1 axis in diabetic nephropathy models, supporting autophagic clearance and mitigating renal injury (Tagawa et al., 2016; Kang et al., 2019).

ULK1 itself represents a key integrative node in this network. Its activation promotes the initiation of autophagy and facilitates the clearance of damaged ER and misfolded proteins, thereby alleviating ER stress and limiting maladaptive UPR signaling (2021; Zhang J.-R. et al., 2024). Notably, AMPK-dependent phosphorylation can activate ULK1 even under conditions of mTORC1 hyperactivation, providing a potential bypass mechanism in diabetes (Wang et al., 2020). Compounds such as geniposide exploit this pathway to enhance autophagic clearance and preserve cellular homeostasis. Beyond bulk autophagy, selective ER turnover through ER-phagy has emerged as an important component of proteostatic control. This process, mediated by receptors such as FAM134B, specifically targets damaged ER regions for lysosomal degradation. In DKD, defective ER-phagy leads to the accumulation of structurally compromised ER, resulting in organelle fragmentation, calcium dysregulation, and sustained stress signaling. This marks a critical transition from adaptive proteostasis to irreversible injury, contributing to tubular atrophy and disease progression. Overall, the balance between adaptive autophagy and its failure represents a key determinant of cell fate in DKD. Once this system collapses, unresolved ER stress and impaired clearance mechanisms reinforce each other, accelerating renal injury.

From a translational perspective, chemical-chaperone strategies (for example, 4-PBA and TUDCA) are used to lower ER proteotoxic load, while selective branch probes are applied to define maladaptive outputs; these interventions currently remain predominantly preclinical in DKD (Cao et al., 2016; Cao et al., 2025; Liu et al., 2025).

### 4.3 Ferroptosis: the UPR-Lipid peroxidation axis

Ferroptosis, first described by Dixon in 2012, is an iron-dependent, non-apoptotic form of regulated cell death driven by the accumulation of lipid peroxides (Dixon et al., 2012). Unlike apoptosis, it primarily disrupts mitochondrial structure rather than nuclear integrity, with features such as reduced mitochondrial volume, increased membrane density, and loss of cristae, while the plasma membrane initially remains intact (Tang et al., 2021). This phenotype reflects its strong dependence on redox imbalance and lipid metabolism. In DKD, ferroptosis has emerged as a key contributor to injury in both podocytes and tubular epithelial cells.

A central step in this process is the failure of the SLC7A11-GPX4 antioxidant system. Experimental models and human DKD samples consistently show reduced expression of SLC7A11 and glutathione peroxidase 4 (GPX4) (Kim et al., 2021; Wang et al., 2023). SLC7A11 supports cystine uptake for glutathione (GSH) synthesis, while GPX4 uses GSH to detoxify lipid hydroperoxides within membranes. Disruption of this axis depletes intracellular GSH and inactivates GPX4, rendering cells unable to neutralize lipid peroxides. Under hyperglycemic conditions, increased ROS and malondialdehyde (MDA), combined with reduced superoxide dismutase (SOD) activity and depleted GSH, further accelerate lipid peroxidation (Shah et al., 2013). Given the intrinsic vulnerability of podocytes to oxidative stress, this antioxidant collapse directly drives structural damage and ferroptotic cell death. Mechanistically, ferroptosis in DKD is closely linked to ER stress and UPR signaling, forming an integrated ER-mitochondria-lipid axis. The ER, as a major site of lipid biosynthesis, generates polyunsaturated fatty acid (PUFA)-containing phospholipids that serve as substrates for peroxidation. Persistent ER stress increases ROS production and disrupts redox balance, creating a permissive environment for ferroptosis. At the same time, maladaptive UPR signaling, particularly through the PERK-ATF4-CHOP pathway, suppresses SLC7A11 expression, limiting cystine uptake and depleting intracellular GSH (Yousaf et al., 2025). This compromises GPX4 activity and weakens the cell’s ability to neutralize lipid peroxides.

Beyond transcriptional regulation, ER stress also promotes ferroptosis through calcium dysregulation and mitochondrial dysfunction. Excessive calcium release from the ER enhances mitochondrial ROS generation, which fuels iron-dependent Fenton reactions and lipid radical formation. In parallel, MAPK pathways, particularly JNK and p38, amplify oxidative stress and inflammatory signaling, reinforcing the ferroptotic cascade. Thus, ferroptosis in DKD arises from the convergence of impaired antioxidant defense, lipid peroxidation, and sustained UPR activation.

Within this network, peroxiredoxin 6 (Prdx6) has emerged as an important endogenous regulator of redox balance and ferroptosis. Through its peroxidase and phospholipase A2 activities, Prdx6 reduces oxidative stress and modulates membrane phospholipid turnover. In diabetic conditions, its upregulation restores GSH levels, limits lipid peroxidation, and preserves podocyte integrity (Zhang et al., 2021). Mechanistically, Prdx6 supports GPX4 and SLC7A11 function while dampening upstream stress pathways, including MAPK signaling and maladaptive UPR activation along the PERK-CHOP axis (Qiu et al., 2026). This positions Prdx6 as a key modulator at the intersection of ER stress and ferroptotic signaling. Despite these advances, several aspects of ferroptosis in DKD remain unresolved. The contribution of ER-associated lipid remodeling enzymes, such as ACSL4, to ferroptotic susceptibility requires further clarification. In addition, iron-handling pathways, including ferritinophagy-mediated release of labile iron, represent an emerging link between ER stress and ferroptosis. Addressing these gaps will be important for developing targeted strategies to modulate ferroptosis and limit renal injury in DKD.

In branch-resolution experiments, ATF6-pathway inhibitors such as Ceapin-A7 are used as mechanistic probes to distinguish adaptive buffering from stress-amplifying outputs; in DKD, these data should be interpreted as early-stage translational evidence (Zhang et al., 2023; Liu et al., 2025).

### 4.4 Pyroptosis: the UPR-inflammasome interface

Pyroptosis is a pro-inflammatory form of regulated cell death that links metabolic stress to the chronic inflammation observed in DKD. Unlike apoptosis, which is largely immunologically silent, pyroptosis is characterized by rapid cell swelling, membrane rupture, and release of pro-inflammatory cytokines such as IL-1β and IL-18, along with damage-associated molecular patterns (DAMPs). In the diabetic kidney, this process is primarily mediated by Gasdermin D (GSDMD). Upon cleavage by inflammatory caspases, the GSDMD N-terminal fragment forms membrane pores, disrupting ionic balance and leading to osmotic lysis in podocytes and tubular epithelial cells (Lin et al., 2020).

Crosstalk between the UPR and pyroptosis is largely driven by the IRE1α–TXNIP–NLRP3 axis. Under chronic glucolipotoxic stress, sustained activation of IRE1α promotes upregulation of thioredoxin-interacting protein (TXNIP), in part through regulated RIDD of inhibitory microRNAs. TXNIP then dissociates from thioredoxin and directly binds NLRP3, promoting inflammasome assembly. This activates caspase-1, which both matures IL-1β and cleaves GSDMD, initiating pyroptosis (Shahzad et al., 2016). Through this pathway, ER stress signaling extends beyond proteostasis, translating metabolic imbalance into a robust inflammatory response that drives immune activation and renal injury (Oslowski et al., 2012; Liu et al., 2021).

In high-glucose podocyte models, oxymatrine has been reported to attenuate NLRP3 inflammasome-dependent pyroptotic injury through SIRT1/NF-κB modulation, supporting pharmacologic tractability of this axis (Xie et al., 2024; Cao et al., 2025; Ouyang et al., 2025).

The PERK-CHOP pathway further amplifies this response. CHOP upregulates key inflammasome components, including NLRP3 and caspase-11, thereby lowering the activation threshold for pyroptosis (Cao et al., 2025). At the same time, ER stress disrupts calcium homeostasis, increasing mitochondrial calcium uptake and promoting mitochondrial ROS (mtROS) production. These mtROS serve as a secondary signal for NLRP3 activation, reinforcing inflammasome activity. Together, TXNIP-driven assembly and mtROS-mediated activation establish a feed-forward loop in which ER stress and mitochondrial dysfunction sustain inflammation and cell death (Oslowski et al., 2012).

Despite these insights, several aspects of pyroptosis in DKD remain unresolved. The extent to which pyroptotic cell rupture propagates injury to neighboring cells, the so-called bystander effect, requires further investigation. In addition, the roles of human inflammatory caspases, particularly caspase-4 and caspase-5, in sensing ER-derived danger signals remain poorly defined. Determining whether selective modulation of UPR signaling can attenuate TXNIP-NLRP3 activation without compromising adaptive proteostasis remains an important goal for the development of targeted anti-inflammatory strategies in DKD.

### 4.5 Necroptosis: the UPR-driven programmed necrosis

Necroptosis is a regulated, caspase-independent form of cell death that combines the signaling control of apoptosis with the morphological features of necrosis, including cell swelling, organelle dysfunction, and plasma membrane rupture. It is executed by the necrosome complex, composed of receptor-interacting protein kinase 1 (RIPK1), RIPK3, and mixed lineage kinase domain-like protein (MLKL) (Pasparakis and Vandenabeele, 2015). Upon activation, MLKL is phosphorylated, oligomerizes, and translocates to cellular membranes, where it disrupts membrane integrity, leading to ionic imbalance and cell lysis. The release of intracellular contents, including damage-associated molecular patterns (DAMPs), makes necroptosis highly pro-inflammatory (Sun et al., 2012).

In DKD, necroptosis has emerged as an important contributor to renal injury, particularly in tubular epithelial cells, where it is associated with tubular damage and interstitial fibrosis. This pathway becomes especially relevant when apoptotic signaling is impaired or when strong inflammatory stimuli such as TNF-α are present, allowing necroptosis to act as an alternative death program that amplifies tissue injury (Linkermann and Green, 2014).

Crosstalk between the UPR and necroptosis provides a direct link between chronic metabolic stress and inflammatory cell death. Persistent ER stress activates PERK and IRE1α, creating conditions that favor necrosome formation. The PERK-eIF2α-CHOP axis contributes by upregulating necroptotic mediators, including RIPK1 and RIPK3, thereby lowering the threshold for pathway activation. In parallel, the IRE1α-TRAF2 axis engages downstream kinases, including JNK, which can facilitate RIPK3 phosphorylation and promote necrosome assembly. Through these mechanisms, sustained UPR signaling effectively primes renal cells for necroptosis in response to inflammatory cues.

This process is further amplified by calcium dysregulation and oxidative stress. Maladaptive UPR signaling drives sustained calcium release from the ER, leading to mitochondrial calcium overload and increased mitochondrial ROS (mtROS) production. Elevated ROS enhances necroptotic signaling, as both RIPK3 activation and MLKL phosphorylation are sensitive to redox conditions (Zhang T. et al., 2016). The combined effects of ER-derived calcium flux and mitochondrial ROS establish a feed-forward loop that stabilizes necrosome activation and promotes membrane rupture. This mechanism likely contributes to the rapid and extensive tubular cell loss observed in advanced DKD, which cannot be fully explained by apoptosis alone (Belavgeni et al., 2020; Zhang et al., 2025).

In proximal tubular epithelial cells, where metabolic demand and exposure to hyperglycemic stress are high, this UPR-necroptosis axis appears particularly prominent (Yu et al., 2023). Increased MLKL activation and membrane translocation correlate with tubular injury and nephron loss, underscoring necroptosis as a key driver of disease progression at later stages. Pharmacological inhibition of RIPK1, such as with necrostatins, represents a potential strategy to limit necroptosis-driven injury. However, whether modulation of UPR signaling can selectively restrain MLKL activation without compromising essential proteostatic functions remains an important question for future investigation (Liu et al., 2025). The summary of these cell death pathways in DKD is presented in Table 1.

**TABLE 1:** Crosstalk mechanisms between UPR and cell death pathways in DKD.

Cell death pathway	Dominant UPR axis	Representative pharmacological modulators & mechanisms	Core DKD relevance	Key references
Apoptosis	PERK-eIF2α-ATF4/CHOP	PERK-oriented branch tools (e.g., GSK2656157) and Bcl-2-axis protective agents (e.g., wogonin) are used to reduce CHOP-biased apoptotic output in preclinical settings	Persistent UPR stress promotes intrinsic apoptotic commitment in DKD renal cells; Reduce progressive podocyte/tubular cell loss	Kaufman, 2002; Cybulsky (2010); Oslowski et al. (2012); Alsamri et al. (2022); Liu et al. (2025)
Autophagy/ER-phagy failure	IRE1α/PERK imbalance with defective clearance	Chemical chaperones (4-PBA, TUDCA) and ER-phagy restoration strategies are used to reduce proteotoxic load and improve clearance capacity	Impaired autophagic and reticulophagic flux sustains ER injury loops; Interrupt chronic ER stress-amplification loops	Wagner and Nebreda (2009); Tabas and Ron (2011); Lerner et al. (2012); Kang et al. (2019); Liu et al. (2025)
Ferroptosis	PERK-ATF4-CHOP stress bias	Redox-preserving strategies plus upstream UPR-dampening interventions are translational candidates for limiting ferroptotic susceptibility; Ceapin-A7 is used as an ATF6-branch probe in branch-resolution studies	Lipid peroxidation and antioxidant collapse increase ferroptotic vulnerability in DKD; Limit oxidative membrane injury and nephron damage	Oslowski et al. (2012); Liu et al. (2016); Liu et al. (2025); Tagawa et al. (2016); Hetz and Papa (2018); Sang et al. (2022); Jin et al. (2024)
Pyroptosis	IRE1α stress signaling and CHOP-linked priming	Anti-inflammasome modulation (e.g., oxymatrine in high-glucose podocyte models) and upstream UPR modulation are complementary candidates	UPR-associated inflammasome activation drives inflammatory renal-cell injury; Reduce inflammatory cell-death amplification	Dixon et al. (2012); Wang et al. (2020); Wang et al. (2023); Alsamri et al. (2022), Oe and Vallon (2022); Jin et al. (2023)
Necroptosis	Stress-amplified RIPK axis coupling	RIPK-pathway inhibition (e.g., necrostatin-type approaches) is mechanistically plausible, with DKD translation still evolving; MiS1 is a SOCS1-mimetic translational candidate	Necroinflammatory signaling contributes to DKD progression and tubular epithelial/interstitial injury programs; Potentially reduce late-stage tubulointerstitial injury	Shah et al. (2013); Shahzad et al. (2016); Liu et al. (2025); Yousaf et al. (2025); Aguilar-Cartes et al. (2026)

Abbreviations in modulators: TUDCA, tauroursodeoxycholic acid; MiS1, SOCS1 mimetic peptide.

Representative delivery-focused translational examples are discussed below.

## 5 Targeted therapeutic strategies and translational opportunities in DKD

To align with the pharmacological scope of this journal, we summarize therapeutic strategies by intervention layer rather than by pathway alone.

First, currently used disease-modifying agents such as SGLT2 inhibitors and nonsteroidal mineralocorticoid receptor antagonists remain foundational because they reduce metabolic and inflammatory load entering renal cells, thereby indirectly attenuating chronic ER stress pressure (Mori et al., 2021; Neuen et al., 2024). However, residual intracellular stress signaling often persists despite hemodynamic and metabolic optimization (Ruiz-Ortega et al., 2020; Liu et al., 2025).

Second, direct ER-stress and UPR-targeted strategies provide mechanistic precision (Liu et al., 2025). In diabetic kidney contexts, TUDCA-containing approaches and related chemical-chaperone concepts have shown renoprotective and stress-attenuating signals in preclinical work (Cao et al., 2016; Zhang J. et al., 2016; Sankrityayan et al., 2023). For branch-selective tools, direct DKD clinical evidence remains limited and should be interpreted as hypothesis-generating rather than practice-changing, with current support still dominated by preclinical branch-level modulation studies (Karekezi et al., 2026).

Third, death-pathway-directed pharmacology is an important translational layer (Wang J. et al., 2025). Recent DKD evidence supports targeting ferroptotic redox collapse, UPR-linked pyroptotic inflammasome signaling, and RIPK-associated necroinflammatory programs as complementary strategies in advanced disease states (Chen et al., 2018).

Fourth, cell-selective delivery is likely to be an important determinant of clinical translation (Zhu et al., 2026). Recent high-impact DKD delivery studies report podocyte-targeted and glycopolymersome-based platforms that improve intrarenal target engagement while aiming to limit systemic off-target exposure (Zhang J. et al., 2024; Guo et al., 2025). At the same time, these studies highlight persistent delivery constraints, including filtration-compartment barriers, cell-type uptake heterogeneity, and unresolved long-term biodistribution/safety questions (Zhu et al., 2026).

## 6 Limitations and future perspectives

Despite profound advances in our understanding of the UPR-Cell Death axis in DKD, several critical challenges remain, hindering the transition from bench to bedside. Addressing these gaps is essential for the development of the next-generation of “proteostatic” therapies. A primary limitation in current DKD research is the heavy reliance on specific rodent models, such as the BTBR ob/ob and STZ-induced mouse models (Aguilar-Cartes et al., 2026). While these models effectively replicate hyperglycemia and certain UPR signatures, they often fail to capture the full hemodynamic and structural complexity of human diabetic nephropathy. Conversely, targeted genetic studies such as podocyte specific IRE1α deletion provide mechanistic support for branch-protective signaling, but they do not resolve model-translation gaps (Cybulsky et al., 2024). Furthermore, a significant demographic bias exists in preclinical studies, which frequently exclude female cohorts (Sandberg et al., 2015). Given that sex hormones significantly influence renal metabolism and UPR sensitivity, future research must incorporate both sexes to ensure the broad clinical applicability of emerging therapies. The therapeutic potential of novel agents, such as SOCS1 mimetics (MiS1), is immense; however, issues surrounding metabolic stability, systemic toxicity, and targeted delivery remain paramount (Aguilar-Cartes et al., 2026). Developing ligand-directed or nanoparticle-based delivery systems that specifically target glomerular podocytes or proximal tubular cells is a prerequisite for moving these interventions into human clinical trials.

## 7 Conclusion

The dysregulated UPR is no longer viewed as a bystander in DKD; it functions as a central signaling hub that translates chronic metabolic disequilibrium into progressive renal injury (Wang F. et al., 2025). The shift from adaptive to maladaptive UPR signaling marks a critical pathogenic transition in renal cells (Liu et al., 2025).

In the diabetic milieu, sustained PERK-CHOP activation, stress-biased IRE1α signaling, and collapse of cellular quality-control systems intensify proteotoxic and inflammatory injury. This dysregulation drives integrated crosstalk with apoptosis, ferroptosis, pyroptosis, and necroinflammatory injury programs in renal compartments (Chen et al., 2018; Wang et al., 2023; Cao et al., 2025; Liu et al., 2025).

Future DKD therapy should aim to restore proteostasis and UPR resolution capacity rather, than only suppress upstream metabolic stress. Combining established systemic treatments, such as SGLT2 inhibitors, with mechanism-guided UPR modulation strategies and cell selective delivery optimization may help narrow the current therapeutic gap and support future disease-modifying development.
